# Potential determinants of the quantity and duration of COVID-19 outbreaks in geriatric long-term care facilities

**DOI:** 10.1186/s12877-023-04446-4

**Published:** 2023-11-20

**Authors:** Janis Evers, Max Geraedts

**Affiliations:** Institute for Health Services Research and Clinical Epidemiology, Philipps-Universität Marburg, Marburg, Germany

**Keywords:** Geriatric long-term care facilities, Profit status, Registered nurses, Risk factors, Single-occupancy rooms

## Abstract

**Background:**

We analyzed potential factors for the number and duration of COVID-19 outbreaks in nursing homes based on routine and structural data.

**Methods:**

All outbreaks during 03/2020-01/2022 in *N* = 687 of a total of 879 geriatric long-term care facilities (LTCFs) in the Federal State of Hesse, Germany were analyzed using t-tests and logistic regressions in a retrospective cohort study.

**Results:**

Larger LTCFs have more (+ 1.57, *p* = .009) and longer outbreaks (+ 10.04 days, *p* > .001). A higher proportion of registered nurses reduces the number (-0.1, *p* = .036) and duration (-6.02 days, *p* > .001) of outbreaks. Single-bed rooms provide less duration of outbreaks (-4.5, *p* = .004). A higher proportion of infected residents (+ 24.26 days, *p* < .001) and staff (+ 22.98 days, *p* < .001) prolong outbreaks the most. LTCFs in areas with intermediate population density have an increased risk of prolonged outbreaks (OR: 1.537, *p* = .036).

**Conclusions:**

To prevent outbreaks and shorten their duration, LTCFs should increase the proportion of registered nurses and single-bed rooms, and control staff infections.

## Background

The global emergence of the SARS-CoV-2 virus causing the COVID-19 pandemic in March 2020 created a rapidly evolving health threat that has claimed nearly 7 million lives until October 2023 [1]. Particularly in geriatric long-term care facilities (LTCFs) with morbid patients and elderly persons, the situation was tense and confusing in the beginning of the pandemic. So COVID-19 outbreaks were associated with life-threatening outcomes for residents and staff in geriatric LTCFs worldwide, especially in the early stages of the pandemic [2, 3]. The initial lack of protection by vaccination led to a condition that made normal facility operations a danger to residents and staff. Outbreaks led to increased mortality among residents and staff. Variations in the number and severity of outbreaks were evident across residential LTCFs - some LTCFs appeared protected from a severe course despite an outbreak, while other LTCFs experienced more frequent and prolonged outbreaks [4–6].

To develop more appropriate measures to protect against frequent and prolonged outbreaks in the future, potential links between structural characteristics as well as the spread and severity of COVID-19 in these LTCFs need to be analyzed. The research available to date paints a homogeneous picture: the benefits of contact reduction on infectious disease containment are indisputable and were therefore widespread practice at the onset of the pandemic [7, 8]. Although closing homes to visitors led to a decreased risk in visitor-caused infections, the risk from infectious staff remained [9, 10]. Smaller LTCFs had statistically fewer outbreaks but appear to be affected by proportionately larger outbreaks [9, 11–13]. Similarly, research currently sees more outbreaks in LTCFs found in urban areas [14] and in COVID-19 hotspots [14], as well as in private sector LTCFs with chain affiliations [15]. A higher registered nurses’ ratio appears to be associated with better control of outbreaks [16]. Similarly, a higher staff skill level is associated with an increased number of outbreaks, while at the same time being a protective factor after an outbreak [17].

Due to mostly missing evidence from Germany, this study investigates the COVID-19 outbreaks and trajectories in Hessian LTCFs during 2020–2022. The purpose was to identify the association between the size of the LTCFs, the population density in the area where the LTCF is located, profit orientation, registered nurses’ ratio, staff-to-bed ratio, and the level of copayments with the total number and duration of outbreaks, in order to derive protective and risk factors. We hypothesize that better funding of LTCFs and a higher proportion of registered nurse’sregistered nurses’protect against frequent and longer COVID-19 outbreaks. Further, we hypothesize that outbreaks are more common in larger LTCFs due to higher rates of outside contacts.

## Methods

For our analyses we combined four datasets containing secondary data about outbreaks and structural characteristics of 687 of a total of 879 geriatric LTCFs in the Federal State of Hesse, one of 16 German states with a population of 6.3 Million which is 7.6% of Germany’s total population. In Hesse, 66,374 residents live in LTCFs that employ 55,158 staff. The study data covered the period 20.03.2020 to 05.01.2022 and was provided in four datasets by the Care Inspectorate of Hesse based at the Regierungspräsidium Gießen and the AOK-Bundesverband, the biggest health insurance provider in Germany provided data for our study. Merging these data was challenging due to inconsistent identifiers and varying information, made more complex by German spelling variations and outdated operator names. We employed fuzzy matching in Microsoft Excel [18, 19] for name and location alignment and manually verified the results through Google Timeline. LTCFs that could not be confidently identified were removed from the analysis.

The group of predictors for the quantity and duration of outbreaks in nursing homes consists of the size of the LTFCs (total number of beds), the proportion of single rooms, the type of provider (0 = non-profit, 1 = for-profit), the monthly copayments for the Level of Care (LoC) 1 and LoC 2+, the registered nursing ratio (registered nurses/nursing professionals to nursing aids), the population density (low (1), medium (2), high (3)), and the staff ratio (calculated from the total number of staff and the total number of beds). In Germany, LTCFs residents have to make a copayment based on their care level and facility rates, which are set through negotiations with long-term care insurances and local governments [20]. Costs can vary due to factors like staff qualifications, service quality, profit orientation, and regional expenses. If residents can’t afford the fees, social welfare may cover some or all costs. Our study focused on costs for the lowest care level, assuming that basic costs are consistent across levels and increase linearly. We also calculated an average score to represent the proportion of infected residents and staff within each Long-Term Care Facility (LTCF), based on the individual outbreaks reported by the facility to the Care Inspectorate. The outcomes under investigation are the number and duration of COVID-19 outbreaks. Outbreaks are defined by the authorities as at least one active COVID-19 case among residents or staff that was identified in connection with the LTCFs. An individual is considered COVID-19 positive if they tested positive either as the initial case or during an outbreak within the long-term care facility, irrespective of where the test was conducted. An outbreak is considered to be over when there are no active COVID-19 cases remaining in the facility. The outbreak duration was operationalized as the temporal interval commencing with the initial positive test result for either a resident or staff member and concluding with the cessation of the infectious period, defined as the point at which no further positive test results were observed.

Descriptive statistics, frequencies, average values, medians, and standard deviations for the expression of the outcome according to predictor variables are reported once for all included LTCFs and once for LTCFs with an outbreak. To evaluate the influence of different structural features, we dichotomized these based on the median and separately assessed their influence on the outcome. Table 2 shows the median for all predictors and the respective number of LTCFs under and over the median.

Additionally, t-tests are performed to show significant associations between the different parameters, with a significance level set at *p* < .05. For variables with unequal variance, we followed the recommendation by Ruxton, Rasch, and Kubinger and performed Welch tests [9–11]. The equality of variance was proved using the Levene test. Furthermore, logistic regressions were used to calculate the odds ratio (OR) or the occurrence of an over-median expression of outcome through predictor. Logistic regression models were chosen for their inherent independence from the normal distribution assumption concerning residuals. This decision was made because our data did not meet the requirements for linear models. To mitigate the potential for overinterpretation, the variables were dichotomized at the median rather than partitioning at an optimal cutoff value. The models were calculated four times. Model 1 comprises all nursing homes and does not consider infected residents or staff as predictors. Model 2 encompasses all nursing homes and includes infected residents or staff as predictors. Model 3 focuses on nursing homes with at least one outbreak and excludes infected residents or staff as predictors. Model 4 concentrates on nursing homes with at least one outbreak and incorporates infected residents or staff as predictors. For models 1&2, LTFCs without outbreak were assigned a value of zero for the duration and frequency of outbreaks and infected residents and staff. The division into these four models makes it possible to make a general (1&2) and a specific assessment of pandemic management after an outbreak (3&4) in a facility. To investigate potential problems due to multicollinearity between the predictor variables, we conducted Pearson and Kendall-Tau correlation analyses, and can exclude that multicollinearity influenced the results of the regression models.

The data analysis was performed using IBM SPSS and Python with the Pandas 1.4.3 package.

## Results

In our analysis, there are 376 non-profit and 306 for-profit LTCFs. About half of the non- (48.4%) and for-profit (50.7%) LTCFs are in areas with a medium population density. For-profit LTCFs have most of their remaining LTCFs (33.3%) in low-density areas, while non-profit LTCFs are in high-density areas (32.3%). Table 1 show the further distributions of Variables by profit status and population density.

**Table 1:** Average structural characteristics of LTCFs in Hesse by location and ownership

	Non-profit (AVG, *N* = 376)	For-profit (AVG, *N* = 306)	Low pop. density (AVG, *N* = 177)	Middle pop. density (*N* = 339)	High pop. density (*N* = 171)	Overall (AVG)
Number of beds	89.4	88.8	73	90	103	88.9
Single rooms (%)	74.2	53.6	61.5	65.6	66.8	64.8
Copayment € (LoC 1)	2639.1	2338.9	2387.3	2466.6	2716.0	2504.9
Copayment € (LoC 2+)	2434.6	1982.9	2047.4	2172.2	2550.1	2230.2
Staff to bed ratio	0.38	0.38	0.38	0.37	0.39	0.38
registered nurses (%)	52.0	48.1	49.7	49.5	52.3	50.3

*LoC* Level of care; *N* Quantity; *AVG* Average; *Pop.* Population

### Quantity of outbreaks – descriptive statistics

 Table 2 shows in detail the expression of average scores of the number of outbreaks by respective predictor, separated for all LTCFs and Table 3 for LTCFs with at least one outbreak. The average number of outbreaks for all LTCFs was 2.16; if only the homes with at least one outbreak are considered, the number was 2.61. The average number of outbreaks was 1.27 (2.81) times higher in larger LTCFs than in smaller LTCFs (Welch, *p* = .009). LTCFs with a higher proportion of infected staff had an average of 0.77 (2.24) more outbreaks than LTCFs with a lower proportion (Welch, *p* < .001). LTCFs with a higher LoC 1 and LoC 2 + copayment had an average of 0.66 (2.29) and 0.54 (2.44) more outbreaks than LTCFs with a lower copayment (Welch, *p* < .001). The average number of outbreaks started was 0.48 (2.34) higher on average in non-profit than in for-profit LTCFs (Welch, *p* < .001). LTCFs with lower staff ratios had on average 0.41 (2.16) more outbreaks than those with high staff ratios (Welch, *p* = .009). LTCFs with a lower proportion of infected residents had on average 0.28 (2.28) more outbreaks than LTCFs with a higher proportion (Welch, < 0.001). The average number of outbreaks started is 0.1 (2.11) higher on average for LTCFs with a lower registered nurses’registered nurses’ ratio than for LTCFs with a higher registered nurses’ ratio (*p* = .036).

**Table 2:** Expression of average and median scores of the number and duration of outbreaks by respective predictor for all LTCFs

				Number of outbreaks			Duration of outbreaks		
	MD	N	MIS	AVG	Std. dev.	*p*	AVG	Std. dev.	
Number of beds (High)	80	346	8	2.81	1.81	.009	34.72	21.39	<.001
Number of beds (Low)		333		1.54	1.37		24.68	20.81	
Prop. of single rooms (%, High)	71.72	340	8	2.24	1.77	.289	28.81	20.51	.451
Prop. of single rooms (%, Low)		339		2.14	1.71		30.78	22.78	
Non-profit	-	376	5	2.34	1.95	<.001	29.92	21.36	.733
For-profit		306		1.91	1.66		29.35	22.19	
Copayment LoC1 (High)	2507.4	326	35	2.29	2.05	<.001	29.33	21.34	.68
Copayment LoC1 (Low)		326		1.85	1.53		30.04	22.33	
Copayment LoC2+ (High)	2201.8	331	28	2.44	1.99	<.001	29.31	21.1	.711
Copayment LoC2+ (Low)		328		1.91	1.61		29.95	22.69	
Staff ratio (High)	.37	339	9	1.83	1.58	.009	28.43	22.45	.100
Staff ratio (Low)		339		2.16	1.71		31.17	20.68	
Prop. of registered nurses (%, High)	49.5	344	0	2.01	1.81	.036	26.53	21.04	<.001
Prop. of registered nurses (%, Low)		343		2.11	1.68		32.37	22.16	
Infected Residents (%, High)	6.38	344	0	2.28	1.33	< .001	41.56	17.13	<.001
Infected Residents (%, Low)		343		2.56	1.89		17.30	19.04	
Infected Staff (%, High)	3.61	344	0	2.24	1.33	<.001	40.92	17.42	<.001
Infected Staff (%, Low)		343		1.72	1.90		17.94	19.56	

*LoC* Level of care; *N* Quantity, *MIS* Missing values; *AVG* Average; *MD* Median; *Std. dev.* Standard deviation

**Table 3:** Expression of average scores of the number and duration of outbreaks by respective predictor for LTCFs with at least one outbreak

			Number of outbreaks			Duration of outbreaks			
	N	MIS	AVG	Std. dev.	*p*	N	AVG	Std. dev.	*p*
Number of beds (High)	313	0	3.11	1.88	<.001	313	38.39	19.10	<.001
Number of beds (Low)	255		2.01	1.22		254	32.23	17.93	
Prop. of single rooms (%, High)	293	0	2.59	1.64	<.001	293	33.44	20.51	.004
Prop. of single rooms (%, Low)	275		2.64	1.78		275	37.94	19.17	
Non-profit	324	1	2.77	1.83	.012	324	34.61	19.10	.141
For-profit	243		2.41	1.51		243	36.96	18.39	
Copayment LoC1 (High)	273	25	3	1.88	<.001	272	35.02	18.55	.446
Copayment LoC1 (Low)	270		2.24	1.41		270	36.26	19.39	
Copayment LoC2+ (High)	279	19	2.89	1.84	<.001	278	34.78	18.36	.249
Copayment LoC2+ (Low)	268		2.33	1.48		268	36.65	19.59	
Staff ratio (High)	276	567	2.43	1.59	.011	275	34.93	19.81	.380
Staff ratio (Low)	291		2.78	1.79		291	36.31	17.88	
Prop. of registered nurses (%, High)	278	0	2.49	1.69	.096	278	32.83	18.45	<.001
Prop. of registered nurses (%, Low)	290		2.73	1.71		289	38.29	18.81	
Infected Residents (%, High)	344	1	2.28	1.33	<.001	283	41.56	17.13	<.001
Infected Residents (%, Low)	223		2.57	1.79		284	26.49	17.64	
Infected Staff (%, High)	344	1	2.24	1.33	< .001	283	40.29	17.42	<.001
Infected Staff (%, Low)	223		2.64	1.77		284	27.48	17.99	

*LoC* Level of care; *N* Quantity, *MIS* Missing values; *AVG* Average; *MD* Median; *Std. dev.* Standard deviation

Additionally, the analysis was performed for all homes with at least one outbreak. LTCFs with a higher total bed count had 1.1 (3.11) more outbreaks on average (*p* < .001). Higher LoC 1 and LoC 2 + co-pays result in 0.76 (3) and 0.56 (2.89) more outbreaks on average (*p* < .001). Lower numbers of infected staff are associated with 0.29 (2.57) more outbreaks on average (*p* = .001). Non-profit LTCFs experienced an average of 0.36 more outbreaks than for-profit LTCFs (*p* < .001). A lower staff to resident ratio was associated with an increase in outbreaks by 0.35 (2.78) on average (Welch, *p* = .011). A lower proportion of infected residents was associated with 0.29 (2.57) more outbreaks (*p* = .001). A lower number of single-bed rooms was associated with 0.005 (2.64) more outbreaks (Welch, *p* < .001).

### Quantity of outbreaks – logistic regression models

In Model 1 (all LTCFs, without scores of infected individuals), a higher total bed count is identified as a risk factor with an OR of 1.017 (95% CI [1.012, 1.022]) (*p* < .001). Similarly, a higher copayment amount is considered a mild risk factor with an OR of 1.001 (95% CI [1, 1.001]) (*p* = .027).

In Model 2 (all LTCFs, with scores of infected individuals), higher total bed count is confirmed as a risk factor with an OR of 1.017 (95% CI [1.012, 1.023]) (*p* < .001). Conversely, a higher number of infected residents is identified as protective with an OR of 0.983 (95% CI [0.969, 0.998]) (*p* = .030). 

All results for Models 1 and 2 regarding the number of outbreaks can be found in Table 4.

**Table 4:** Odds ratios with 95% confidence intervals for above-median number and duration of outbreaks in LTCFs (models 1 & 2)

		Model 1				Model 2			
	Predictors	95%-CI				95%-CI			
		OR	LCI	UCI	*p*	OR	LCI	UCI	*p*
Number of outbreaks	Number of beds	1.017	1.012	1.022	<.001	1.017	1.012	1.023	<.001
	Prop. of single rooms (%)	1.001	.995	1.008	.650	1.001	.994	1.007	.849
	For-profit (ref. = non-profit)	.815	.545	1.218	.318	.794	.529	1.191	.265
	Medium pop. density (ref. = low pop. density)	1.087	.725	1.63	.686	1.061	.705	1.597	.777
	High pop. density (ref. = low pop. density)	1.066	.630	1.803	.811	1.007	.597	1.714	.981
	Copayment LoC1	1.001	1.0	1.001	.027	1.001	1.0	1.001	.06
	Staff ratio	.566	.101	3.178	.518	.716	.124	4.132	.709
	Prop. of registered nurses	.566	.966	1.007	.197	.984	.964	1.005	.134
	Infected residents (Model 2)					.983	.969	.998	.030
	Infected staff (Model 2)					1.013	.980	1.048	.435
Duration of outbreaks	Number of beds	1.01	1.006	1.014	<.001	1.011	1.006	1.017	<.001
	Prop. of single rooms (%)	.998	.992	1.004	.475	1.004	.996	1.012	.316
	For-profit (ref. = non-profit)	.914	.617	1.353	.653	1.035	.649	1.653	.884
	Medium pop. density (ref. = low pop. density)	1.537	1.029	2.297	.036	2.162	1.281	3.648	.004
	High pop. density (ref. = low pop. density)	1.246	.744	2.087	.403	1.868	.993	3.514	.053
	Copayment LoC1	1.0	.999	1.001	.783	1.001	1.0	1.001	.135
	Staff ratio	3.272	.570	18.788	.184	2.978	.376	23.593	.301
	Prop. of registered nurses	.953	.933	.973	<.001	.950	.926	.974	<.001
	Infected residents					1.059	1.03	1.089	<.001
	Infected staff					1.088	1.023	1.156	.007

*N* = 643, total missing = 44

*Ref*. Reference; *Pop.* Population; *LoC* Level of care; *Prop.* Proportion; *MIS* Missing values;*OR* Odds ratio; *CI* Confidence intervall; *LCI* Lower confidence intervall; *UCI* Upper confidence Interval

In Model 3 (LTCFs outbreak > 0, without scores of infected individuals), both the total number of beds and the level of copayments are considered mild risk factors. The total number of beds has an OR of 1.016 (95% CI [1.01, 1.022]) (*p* < .001), while the level of copayments has an OR of 1.001 (95% CI [1.001, 1.002]) (*p* < .001).

In Model 4 (LTCFs outbreak > 0, with scores of infected individuals), similar to Model 3, the total number of beds and the level of copayments are identified as mild risk factors. The total number of beds has an OR of 1.016 (95% CI [1.01, 1.022]) (*p* < .001), and the level of copayments has an OR of 1.001 (95% CI [1.001, 1.002]) (*p* < .001). All results for Models 3 and 4 regarding the number of outbreaks can be found in Table 5.

**Table 5:** Odds ratios with 95% confidence intervals for above-median number and duration of outbreaks in LTCFs (models 3 & 4)

		Model 3				Model 4			
	Predictors	95%-CI				95%-CI			
		OR	LCI	UCI	p	OR	LCI	UCI	p
Number of outbreaks	Number of beds	1.016	1.1	1.022	<.001	1.016	1.01	1.022	<.001
	Prop. of single rooms (%)	1.0	.993	1.008	.92	1.0	.992	1.007	.901
	For-profit (ref. = non-profit)	1.104	.691	1.763	.679	1.122	.699	1.8	.634
	Medium pop. density (ref. = low pop. density)	1.142	.728	1.791	.563	1.147	.727	1.81	.555
	High pop. density (ref. = low pop. density)	1.397	.754	2.586	.288	1.439	.77	2.689	.254
	Copayment LoC1	1.001	1.001	1.002	<.001	1.001	1.001	1.002	<.001
	Staff ratio	.936	.116	7.553	.951	.879	.105	7.337	.906
	Prop. of registered nurses	.99	.967	1.014	.419	.986	.963	1.01	.261
	Infected residents					.894	.49	1.63	.714
	Infected staff					.598	.33	1.085	.091
Duration of outbreaks	Number of beds	1.007	1.002	1.011	.003	1.009	1.004	1.015	<.001
	Prop. of single rooms (%)	.992	.985	.998	.012	.995	.988	1.003	.257
	For-profit (ref. = non-profit)	.941	.617.	1.436	.778	.922	.571	1.49	.741
	Medium pop. density (ref. = low pop. density)	1.375	.890	2.123	.151	1.761	1.043	2.973	.034
	High pop. density (ref. = low pop. density)	1.385	.789	2.431	.257	1.87	.982	3.561	.057
	Copayment LoC1	1.00	1.00	1.001	.232	1.001	1.0	1.002	.008
	Staff ratio	6.787	.865	54.69	.068	5.519	.568	53.587	.141
	Prop. of registered nurses	.959	.937	.981	<.001	.955	.930	.981	<.001
	Infected residents					1.045	1.023	1.067	<.001
	Infected staff					1.042	.996	1.091	.077

*N* = 542, total missing = 26

*Ref.* Reference; *Pop.* Population; *LoC* Level of care; *Prop.* Proportion; *MIS* Missing values;*OR* Odds ratio; *CI* Confidence intervall; *LCI* Lower confidence intervall; *UCI* Upper confidence Interval

### Duration of outbreaks – descriptive statistics

Table 2 shows the expression of average scores of durations of outbreaks by respective independent variable, separately for all LTCFs and Table 3 for LTCFs with at least one outbreak. When all LTCFs were considered, the average outbreak duration was 29.45 days, and 35.62 days for LTCFs with at least one outbreak. Outbreaks in LTCFs with a higher proportion of infected residents were prolonged by 24.26 (41.56) days more than in LTCFs with a lower proportion (*p* < .001). LTCFs with a higher proportion of infected staff had 22.98 (40.29) days (*p* < .001) of prolonged outbreaks compared to LTCFs with a lower proportion. The average duration in larger LTCFs was 10.04 (34.72) days longer than in smaller ones (*p* < .001). LTCFs with a lower registered nurses’ ratio had outbreaks that lasted on average 5.84 (32.37) days longer than LTCFs with a higher registered nurses’ ratio (*p* < .001).

In addition, an analysis was performed for all homes with an outbreak. LTCFs with a higher proportion of infected residents had outbreaks that were 15.07 (41.56) days longer than LTCFs with a lower proportion (*p* < .001). LTCFs with a higher proportion of infected staff had outbreaks that were 12.81 (40.29) days longer than LTCFs with a lower proportion (*p* < .001).

The average duration of outbreaks was 6.16 (38.39) days longer in larger LTCFs than in smaller LTCFs (*p* < .001). The average duration of outbreaks in LTCFs with a lower registered nurses’ ratio was 5.46 (38.29) days longer than LTCFs with a higher ratio (*p* < .001). Compared to LTCFs with a higher proportion of single-bed rooms, LTCFs with a lower proportion of single-bed rooms had a prolonged duration of 4.5 (37.94) days on average (*p* < .001).

### Duration of outbreaks – logistic regression models

In Model 1 (all LTCFs, excluding scores of infected individuals), an odds OR of 1.537 (95% CI [1.029, 2.297]) indicates that intermediate population density is a risk factor for prolonged outbreaks (*p* = .036) compared to low population density. A higher total bed count is also a mild risk factor with an OR of 1.01 (95% CI [1.006, 1.014]) (*p* < .001). In contrast, a higher skilled worker ratio is a mild protective factor with an OR of 0.953 (95% CI [0.933, 0.973]) (*p* < .001).

In Model 2 (all LTCFs, with scores of infected individuals), an OR of 2.162 (95% CI [1.3, 3.65]) (*p* = .004) indicates that average population density is a risk factor. A higher proportion of infected staff has an OR of 1.09 (95% CI [1.02, 1.6]) (*p* = .007), a higher proportion of infected residents with an OR of 1.06 (95% CI [1.03, 1.09]) (*p* < .001), and a higher total number of beds with an OR of 1.01 (95% CI [1, 1.02]) (*p* < .001) are also risk factors. A higher registered nurses’ ratio is identified as a protective influence with an OR of 0.95 (95% CI [0.93, 0.97]) (*p* < .001).

All results for Models 1 and 2 regarding the duration of outbreaks can be found in Table 4.

In Model 3 (LTCFs outbreak > 0, without scores of infected individuals), a higher registered nurses’ ratio with an OR of 0.959 (95% CI [0.937, 0.981]) (*p* < .001) and a higher proportion of single-bed rooms with an OR of 0.992 (95% CI [0.985, 0.998]) (*p* = .012) are identified as protective effects. A higher total number of beds is considered a mild risk factor with an OR of 1.007 (95% CI [1.002, 1.011]) (*p* = .003).

In Model 4 (LTCFs outbreak > 0, with scores of infected individuals), a higher number of infected residents with an OR of 3.692 (95% CI [2.105, 6. 478]) (*p* < .001) and a higher number of infected staff with an OR of 2.557 (95% CI [1.474, 4.436]) (*p* < .001), as well as an average population density with an OR of 1.681 (95% CI [1.025, 2.757]) (*p* = .039), are identified as risk factors. A higher total number of beds is considered a mild risk factor with an OR of 1.008 (95% CI [1.003, 1.013]) (*p* = .002), while a higher registered nurses’ ratio is a protective factor with an OR of 0.957 (95% CI [0.932, 0.982]) (*p* < .001).

All results for Models 3 and 4 regarding the duration of outbreaks can be found in Table 5. 

## Discussion

Our study identified several characteristics of LTCFs to be associated with the number and duration of outbreaks during the SARS-CoV-19-pandemic in Hesse, Germany. Our findings indicate that larger LTCFs are at higher risk for more frequent and longer outbreaks. For-profit LTCFs had fewer outbreaks than non-profit homes, but this could be due to different testing regimes or test availability. A higher ratio of registered nurses’was associated with fewer and shorter outbreaks, and a higher proportion of infected residents was associated with fewer outbreaks in LTCFs.

Our study confirms findings related to LTCFs’ size [17, 21–23]. Large LTCFs were on average more at risk of more frequent outbreaks when compared to individual independent variables. This effect is seen in both groups studied and is confirmed by all four models. A higher number of outside contacts and thus an increased probability of an outbreak might be an explanation. Similarly, the duration of outbreaks was significantly increased for larger LTCFs in the bivariate statistics. This effect is seen in both groups and is confirmed as a slight risk factor by all models. A larger number of residents may increase the likelihood of protracted outbreaks.

For-profit LTCFs had fewer outbreaks than non-profit homes. The statistical difference between for-profit and non-profit LTCFs may be explained by differences in testing regimes or test availability: Test shortages at the beginning of the pandemic resulted in soaring prices for test kits. LTCFs that did not pay extra for routine testing, for example, and only tested for symptoms, had patchier infection monitoring and fewer outbreaks were found. Therefore, a higher number of outbreaks does not necessarily imply that LTCFs are worse in terms of hygiene, but that they detected more outbreaks. In this regard, Gorges and Konetzka [23] figured out that non-profit homes had more outbreaks, and homes with outbreaks had better access to test kits. This is consistent with the higher number of outbreaks in more expensive LTCFs, which have more resources because of higher co-pays. This should be further investigated through time series analyses with price and availability trends.

We observed prolonged outbreaks in the models 1,2 and 4 with medium population density, but we do not have a substantive explanation for this; further research is needed in this area.

While a systematic review has reported a greater number of outbreaks at higher staff ratios [17], our data suggest that higher staff ratios may be associated with fewer outbreaks. At a higher ratio, infections of staff are more easily compensated for, and therefore individuals with mild symptoms of illness are less pressured to report for work. Shallcross et al. reached a comparable conclusion for LTCFs in England that pay sick leave [24]. Further, the staff ratio is slightly higher in smaller LTCFs, which may also have contributed to this effect. A higher ratio of registered nurses was associated with a lower number of outbreaks in the bivariate statistics for all LTCFs.

Registered nurses are generally more likely to implement protective measures adequately due to their better training. This is also shown by the significantly lower number of outbreak days in LTCFs with a higher registered nurses’ ratio and was already confirmed in a Korean study [16]. The ratio of registered nurses was also higher in small homes, which were associated with fewer outbreaks. However, the effects of skilled worker ratio and staff ratio were not significant in any of our models.

A higher proportion of infected residents was associated with fewer outbreaks in the LTCFs. On the one hand, this can be explained by a few larger outbreaks with a more comprehensive number of residents resulting in short-term immunity. Second, the real duration of larger outbreaks is longer and thus prevented multiple individual outbreaks from occurring during the study period. Furthermore, it can be assumed that large outbreaks are the result of poorer infection monitoring, so that fewer outbreaks were generally detected in these LTCFs. This ‘protective’ influence is also evident in Model 2, where LTCFs with a high proportion of infected staff show the highest number of outbreaks. These findings suggest that infected staff is one of the strongest drivers of COVID-19 in the LTCFs, along with their overall size. Restricting the analysis to those LTCFs with at least one outbreak, the effect reverses, showing fewer outbreaks with a higher proportion of infected staff, but this was not confirmed by any multivariate model. The longer duration of a gradual spread of the virus and the short-term immunization may have resulted in fewer outbreaks, but perhaps also that more infected staff were detected through early testing.

Our study has methodological and content limitations. Data merging was challenging due to inconsistent identification variables and variable information, further complicated by location and spelling issues. Fuzzy matching had its limits, particularly with abbreviations and umlauts. LTCFS that couldn’t be confidently identified were excluded from the analysis. However, we don’t believe this introduced bias, as the exclusions were random. Uniform keys should be assigned to LTCFs for all datasets that go beyond a change of operator to avoid this in the future. Due to the many influencing factors, most significant results have large standard deviations and confidence intervals. To obtain consistent results, the study should be repeated on a larger data set, e.g. in a nationwide comparison or over a longer period. Furthermore, the variance resolution of models 1 and 3 is low. In terms of content, local incidence at the time of the outbreak would have contributed much to this variance elucidation, but so would other structural drivers such as mask compliance, number of vaccinations administered, implementation of spacing rules, visitor rules, new room occupancy, testing regimen, the number of residents per bathroom, organization of the kitchen, cleaning and food service, implementation of group offers, stay in common rooms, external additional offers such as hairdressers and chiropodists, the actual care time and the construction type of the facility, which were all not considered in the analysis due to lack of data. Despite extensive efforts, our study faced limitations due to strict privacy regulations that made specific data description of the resident population in terms of age, gender, or level of autonomy inaccessible. We could only conjecture the reasons for the many effects emanating from registered nurses or set up links from the literature. Qualitative research could help to better understand the differences between registered nurses and other staff in managing pandemics. If necessary, best practices should be developed. Furthermore, the distinction between for-profit and non-profit LTCFs is not always purposeful since both profit status groups must generate and reinvest profit. A more nuanced approach to classifying LTCFs may be warranted, considering factors such as stock exchange listings, private equity affiliations, non-profit chains with an increased profit motive, and other institutional characteristics.

## Conclusion

Large LTCFs should be aware of more frequent outbreaks due to the increased external contacts and update them, as necessary. A higher staff quota may contribute to better sickness cover, and with denser staffing levels, extra staff can be assigned to corona rooms. The protective effects of a higher registered nurses’ ratio on the number and duration of outbreaks are noticeable, as no big difference in this ratio was expected based on the statutory ratio. Further research is needed to investigate the relationship between the impact of registered nurses on infection prevention measures. The prolonged duration of outbreaks in areas with medium population density cannot be explained and should be further investigated. Single rooms likely reduce the duration of outbreaks due to better isolation possibilities; the proportion of such rooms should therefore be increased.

## Data Availability

The data that support the findings of this study are available from Hessische Betreuungs- und Pflegeaufsicht but restrictions apply to the availability of these data, which were used under license for the current study, and so are not publicly available. Data are however available from the authors upon reasonable request and with permission of Hessische Betreuungs- und Pflegeaufsicht.
